# Supplementary material for: Supernova dust destruction in the magnetized turbulent ISM
Source: Nat Commun. 2024 Feb 28;15:1841. doi: 10.1038/s41467-024-45962-0 (PMC10901883; doi:10.1038/s41467-024-45962-0)
Supplement: Supplementary file 1 — Supplementary Information [file 41467_2024_45962_MOESM1_ESM.pdf]

Supernova dust destruction in the magnetized turbulent ISM

Florian Kirchschlager[1,2†], Lars Mattsson[3*†] and Frederick A. Gent[4,5†]

[1]Physics and Astronomy, Ghent University, Krijgslaan 281-S9, Ghent, 9000, Belgium.
[2]Physics and Astronomy, University College London, Gower Street, London, WC1E 6BT, UK.
[3]Nordita, KTH Royal Institute of Technology and Stockholm University, Hannes Alfvéns väg 12, Stockholm, SE-106, Sweden.
[4]Astroinformatics, Computer Science, Aalto University, PO Box 15399, Espoo, FI-00076, Finland.
[5]School of Mathematics, Statistics and Physics, Newcastle University, Newcastle, NE1 7RU, UK.

*Corresponding author(s). E-mail(s): lars.mattsson@su.se;
Contributing authors: florian.kirchschlager@ugent.be; frederick.gent@aalto.fi;
†These authors contributed equally to this work.

This Supplementary file contains:

Supplementary Figure 1: Temporal evolution of the gas for an explosion in the moderate density region
Supplementary Figure 2: Temporal evolution of the gas for an explosion in the low density region
Supplementary Figure 3: Temporal evolution of the dust for an explosion in the moderate density region
Supplementary Figure 4: Temporal evolution of the dust for an explosion in the low density region
Supplementary Figure 5: Scatter plots of gas and dust maps for the model NM
Supplementary Figure 6: Scatter plots of gas and dust maps for the model BM
Supplementary Figure 7: Mean gas density as function of time
Supplementary Figure 8: Dust destruction fractions

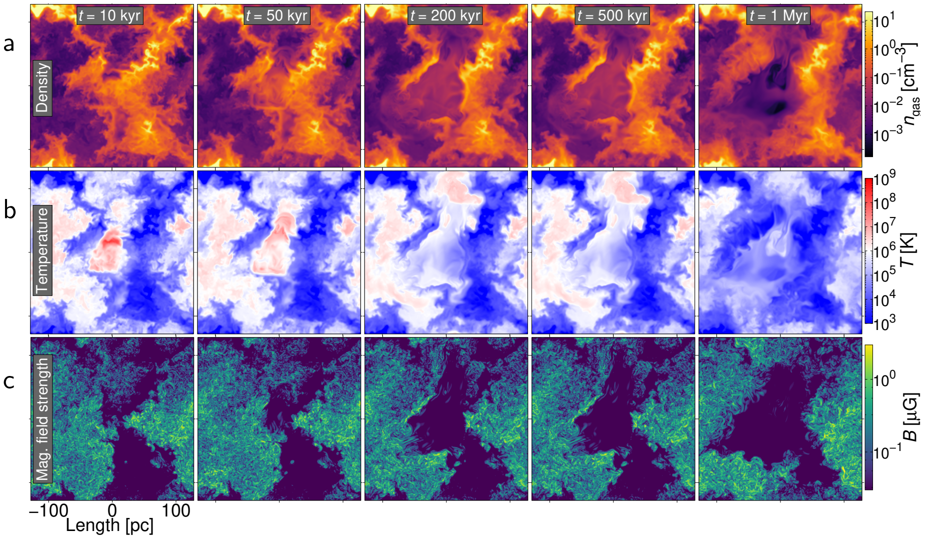

Supplementary Figure 1: Temporal evolution of the gas. a Gas density. **b** Gas temperature. **c** Magnetic field strength and direction. The snapshots are at $t = 10\,$kyr, $50\,$kyr, $200\,$kyr, $500\,$kyr, and $1\,$Myr (compare with Figure 1 in the main paper). The SN explodes in a moderate density region (center of the box; model BM).

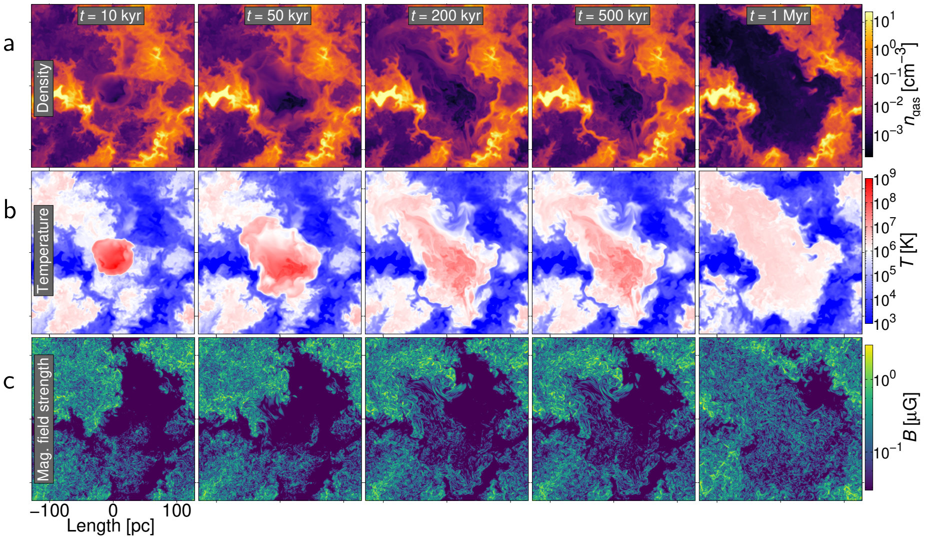

Supplementary Figure 2: Same as Supplementary Figure 1, but for an explosion in the low density region (model BL).

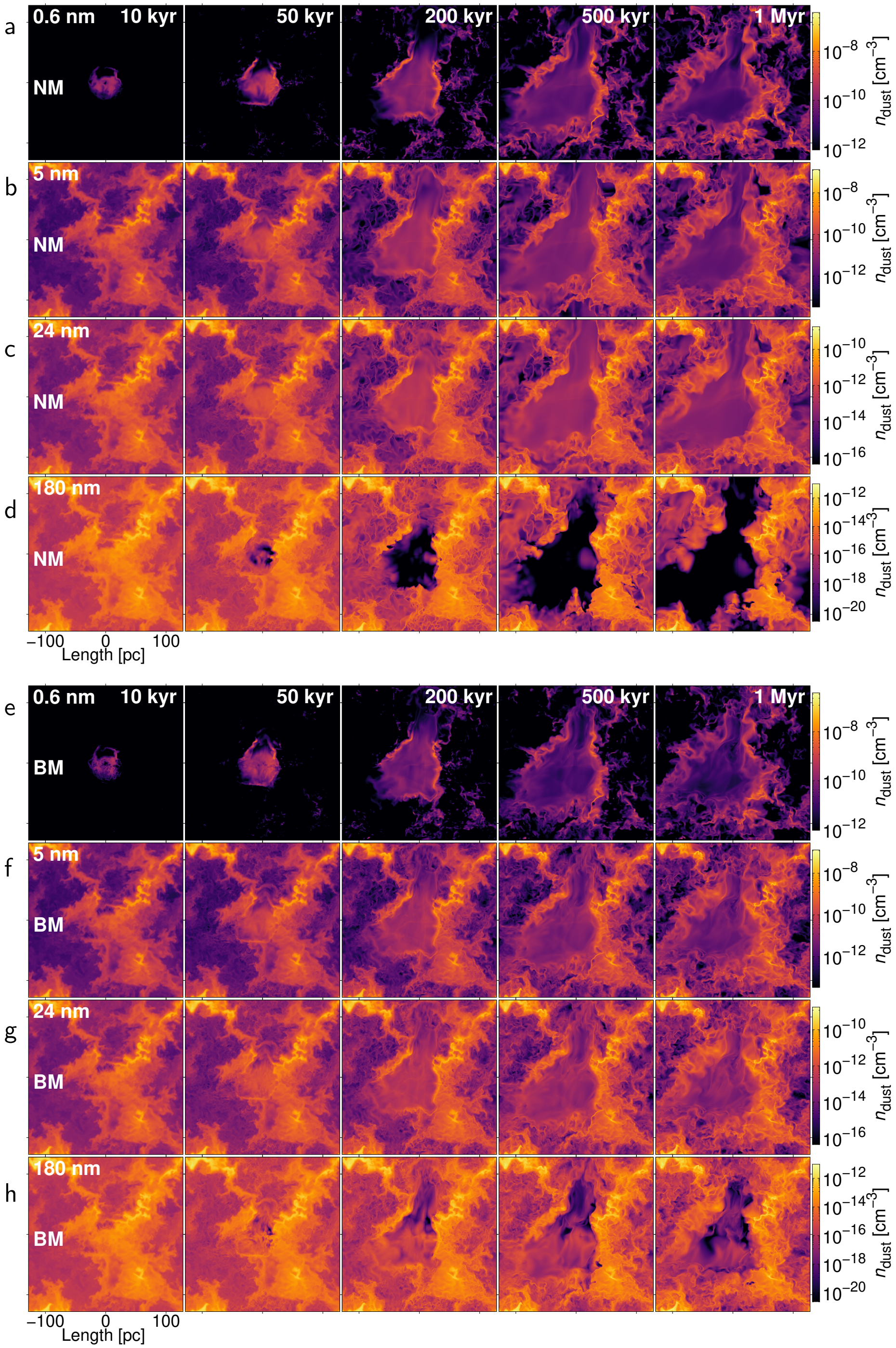

Supplementary Figure 3: Temporal evolution of the spatial dust density when the SN explodes in the moderate density region. (compare with Figure 3 in the main paper). **a - d** Dust grains without magnetic field effects (model NM). **e - h** Lorentz force is included in the calculations (model BM). The first, second, third, and fourth row of each block show the distribution of 0.6, 5, 24, 180 nm grains, resp.; the colour scale is fixed for each row.

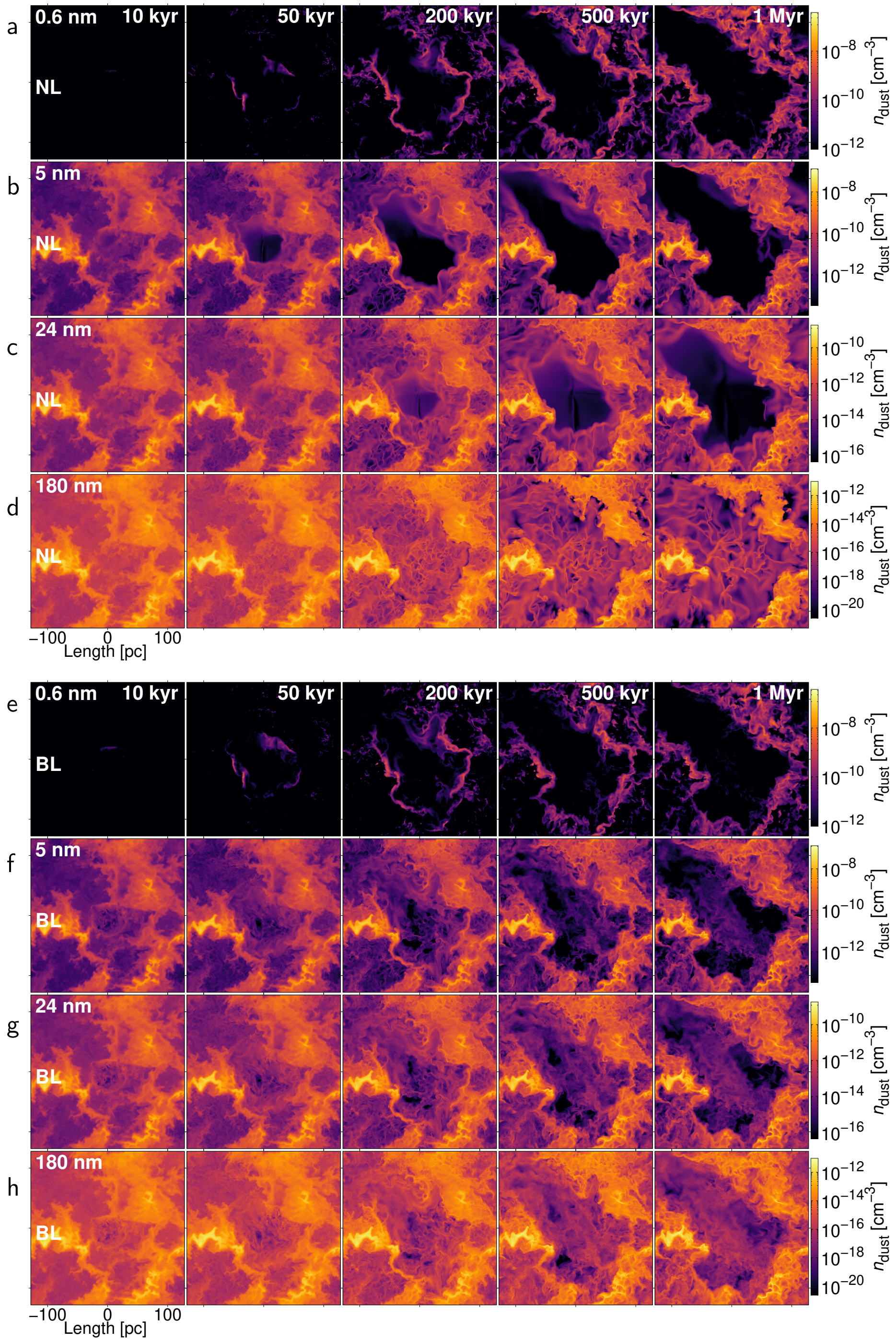

Supplementary Figure 4: Same as Supplementary Figure 3, but for an explosion in the low density region. **a - d** Model NL. **e - h** Model BL.

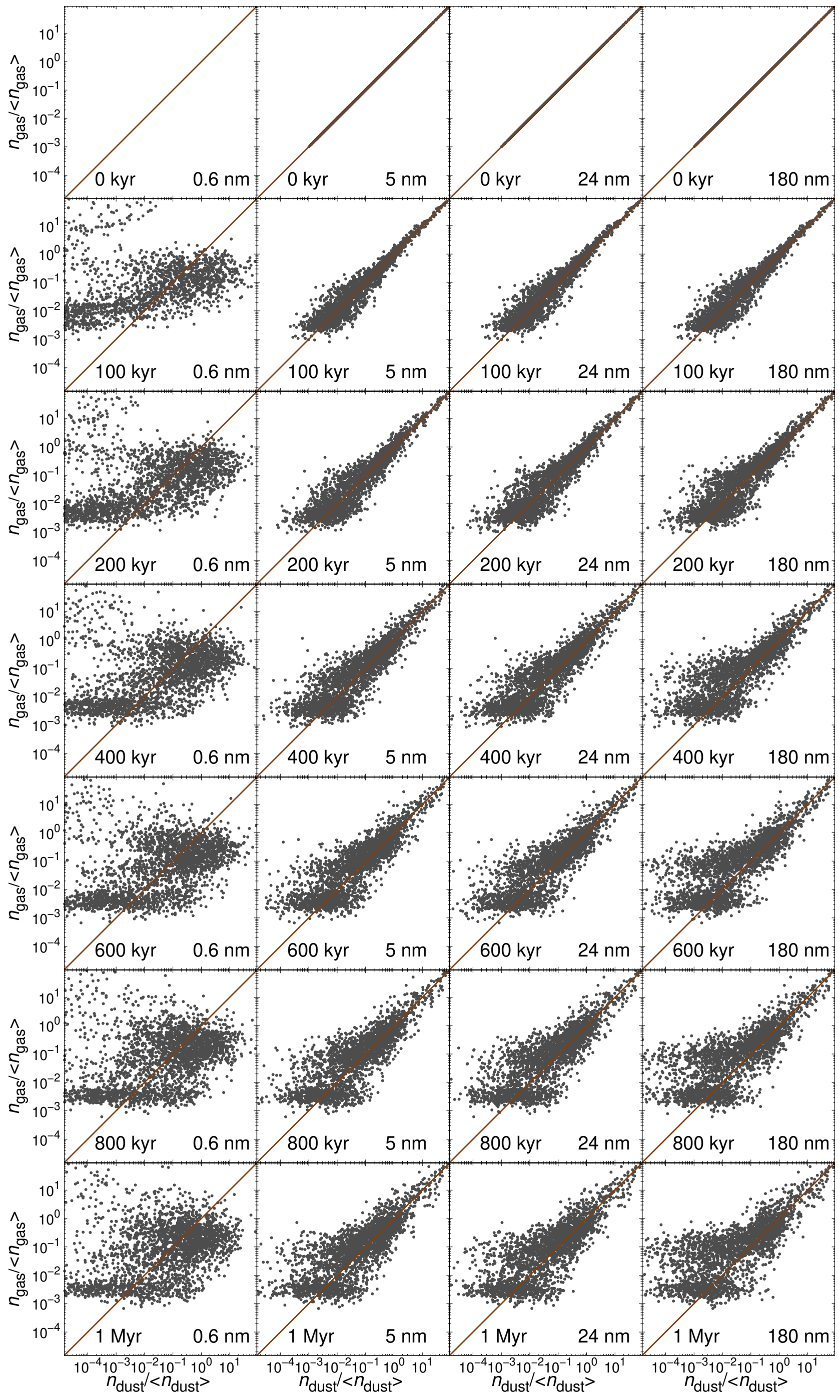

Supplementary Figure 5: Scatter plots of the normalized gas density $n_{\mathbf{gas}}/<n_{\mathbf{gas}}>$ and dust density $n_{\mathbf{dust}}/<n_{\mathbf{dust}}>$. The plots are for maps at timesteps between 0 and 1 Myr after the explosion of the SN in the moderate density region (model NM) and four grain sizes (columns). The brown line shows the case where gas and dust density are proportional to each other (perfect coupling).

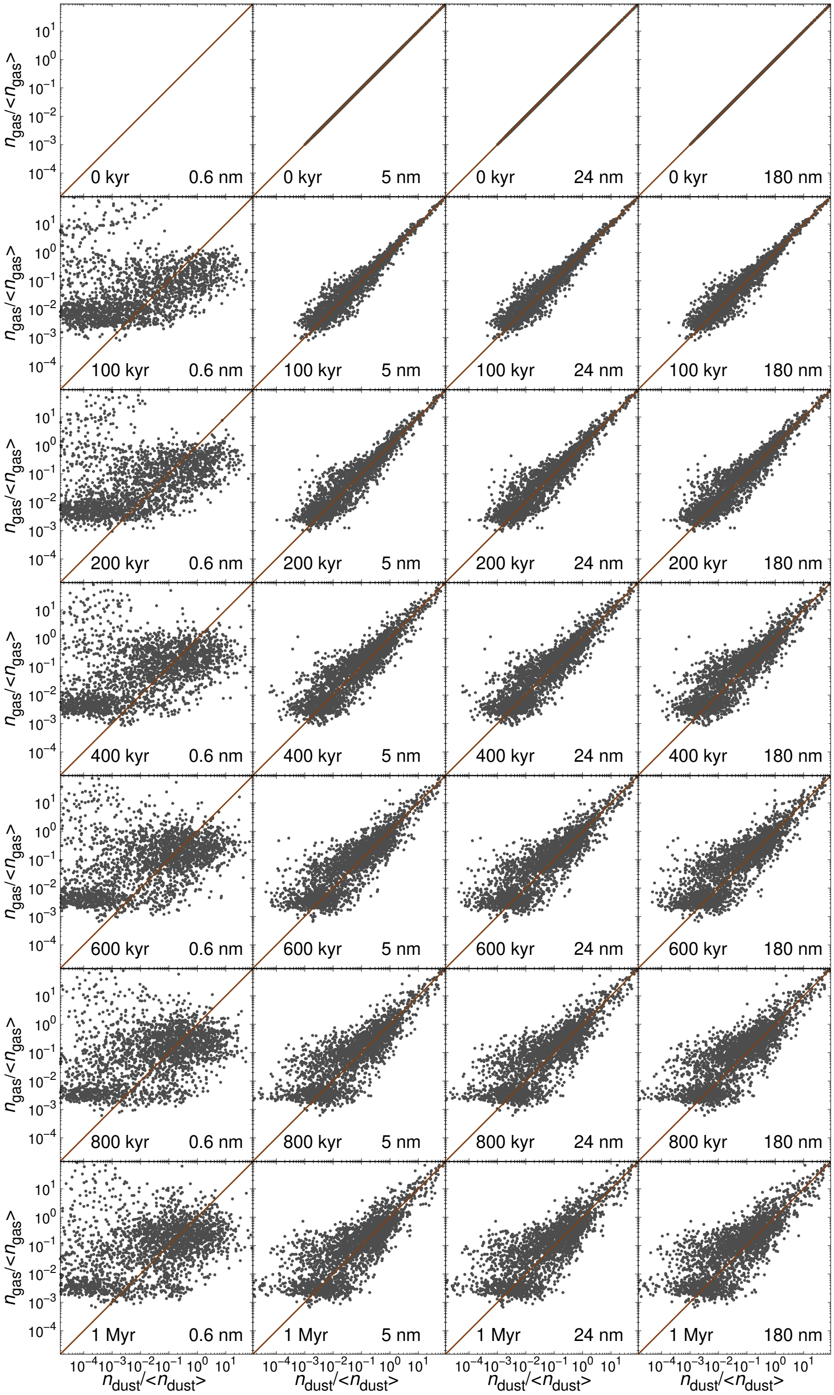

Supplementary Figure 6: Same as Supplementary Figure 5, but for the model BM.

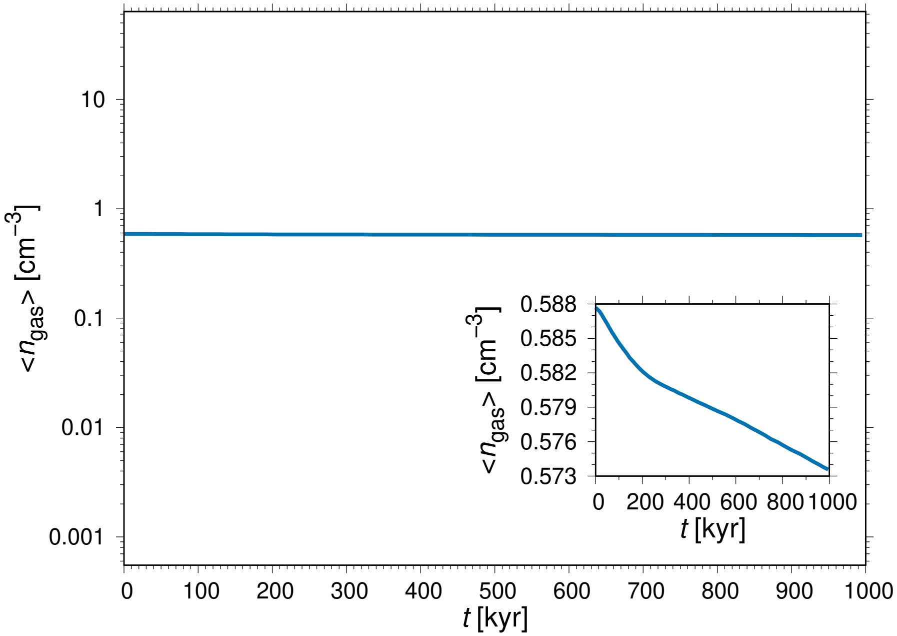

Supplementary Figure 7: Mean gas density $<n_{\mathbf{gas}}>$ of the slice as a function of time. The range of the mean gas density spans from the minimum to the maximum gas density at a single grid cell at the first snapshot ($t = 0$). The plot indicates that there is little change over time compared to the initial inhomogeneities. The inset shows the mean gas density on a smaller range, indicating that the mean gas density has a monotonic decay.

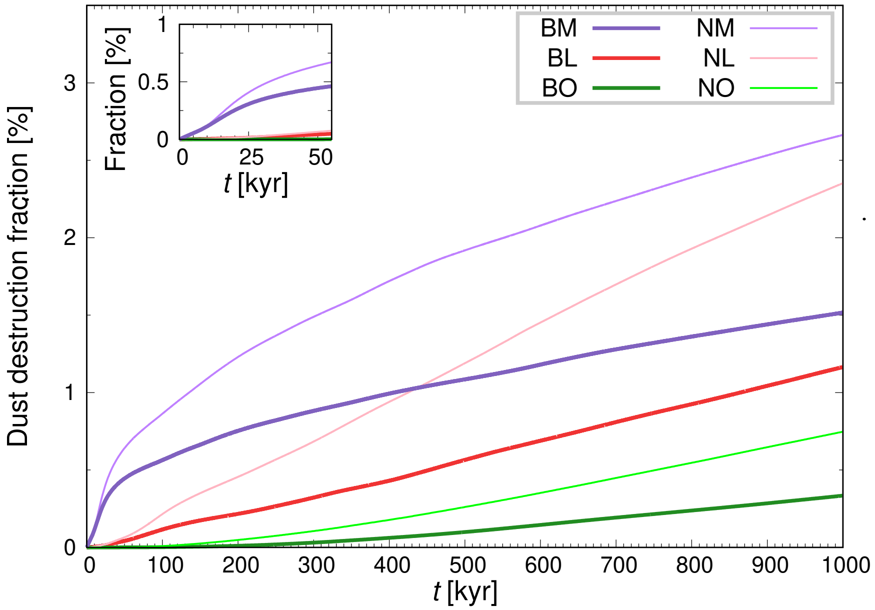

Supplementary Figure 8: Dust destruction fractions as a function of time. The inset shows the destruction fraction within the first 50 kyr. We note that these fractions show the ratio of the destroyed dust mass to the total dust mass in the entire 3D domain. As destruction by the blast wave is ongoing only in a part of this domain, the presented fractions serve only as a lower limit.
